# Clinical Lectures and Cases

**Published:** 1851-05

**Authors:** N. S. Davis

**Affiliations:** Prof. of Principles and Practice of Medicine in Rush Medical College, and one of the Physicians to the Hospital


### THE NORTH-WESTERN MEDICAL AND SURGICAL JOURNAL.

VOL. IV.] MAY, 1851. [No. 1.

### Part 1.—Original Communications.

ARTICLE I.

*Clinical Lectures and Cases in the Medical Wards of the Illinois General Hospital.* By N. S. DAVIS, M. D., Prof. of Principles and Practice of Medicine in Rush Medical College, and one of the Physicians to the Hospital. Reported by B. F. WHITE, Interne.

*(Continued.)*

CASE 2d. E. B., Irish laborer, aged about 20 years, admitted into the Hospital this day, Jan. 10th. Visited by the attending physician accompanied by his class at 5 o'clock P. M.

You perceive, said the Doctor, that the position of the patient is dorsal with a slight inclination to the right side; his face is deeply flushed, but of a purplish or dingy red color, with something of a bloated aspect; his expression dull and inactive; his skin dry, harsh, and warmer than natural; his chest is broad and full, but his breathing short, difficult, and painful; his cough appears frequent and productive of deep seated pain, and soreness in the central part of the right half of the chest, with a pretty copious liquid, and reddish or bloody expectoration, which you will see by looking in this vessel, is pretty homogeneous, and not simply streaked with blood. It is tenacious, however, and of a veinous rather than arterial redness. His tongue, you perceive, is covered with a pretty thick yellowish fur, quite dry, and slightly redened along the edges and tip; his pulse is little more than 100 per minute, not full, but easily compressed; thirst moderate; urine scanty and high colored; and his bowels rather tympanitic, with loose watery evacuations. The patient says he was seized with a sense of chillness, pain in his chest, difficult breathing, and cough, while at work four days since, which soon became so severe as to compel him to return home and take his bed. This chillness soon ceased and gave place to a head-ache, thirst, and all of the symptoms which we have just passed in review. He has had no medical attention, but has taken two or three doses of physic, (castor oil and epsom salts), which accounts, in part, at least, for the looseness of his bowels at the present time.

This brief history of the case, with the prominent symptoms which we have passed in review, is sufficient to direct our attention to the thoracic viscera as the special seat of disease.

Indeed, all the general symptoms sufficiently characterize the case, as one which is commonly styled *lung fever*, or *winter fever*, in many parts of the country, and which in some sections is exceedingly prevalent and fatal. But to gain rational and accurate indications of treatment, we must ascertain more definitely the location, extent, and stage of the disease. As I lay the chest bare, you observe the left side moves much more freely with each inspiration than the right. Each of you can detect the difference in the resonance, as I compare the one side with the other, by percussion; there being pretty decided dullness over the upper, and the greater share of the middle lobe of the right lung. The dullness seems greatest about two inches above the nipple. By extending the percussion over the abdominal viscera we find no indications of enlargement of the solid viscera, but there is a general tympanitic condition.

Returning to the chest I apply my ear to the left side and hear nothing but an exaggerated or puerile respiration.

Over the infra clavicular region of the right side, the respiration is distinctly bronchial and somewhat intermixed with a coarse mucous rattle or rhonchus, and the voice is decidedly bronchial. Over the upper part of the mammary region and bordering on the axilla, the fine crepitant râle is very distinct. I find no morbid sounds over the remaining part of the chest. The cardiac sounds are normal, the impulse though frequent is only of moderate force. Such are the physical signs elicited by percussion and auscultation, and they indicate simply an unnaturally solid condition of the upper, and part at least, of the middle lobes of the right lung. But if taken in connection with the general symptoms, particularly the fever, deep seated pain in the chest, oppressed respiration, cough, and bloody expectoration, there is left no difficulty or doubt in reference to a proper *diagnosis.* The solidity indicated by the dullness and bronchophony, is simply an early stage of *hepatization* of the right lung, while the crepitant râle heard at the lower and lateral margin of the dulness, shows that inflammation in its first stage exists, and is probably still extending to other portions of the lung.

*Diagnosis.* We have then, gentlemen, a case of acute Pneumonia, in the fourth day of its progress, involving the upper and middle lobes of the right lung, in a considerable portion of which the disease has passed into the stage of infiltration, more commonly called *hepatization.* But this is not all.

For the appearance of the tongue, the tympanitic state of the abdomen, and the loose or watery evacuations, show plainly that the pneumonia is complicated with irritation, if not a low grade of inflammation of the mucous membrane of the alimentary canal. Before calling your attention to the treatment, however, I wish you to mark well the differences between this case and that of T. Y., discharged a few days since. (See March No. of Journal, Ed.) The principle disease in both was acute pneumonia, and both were admitted at about the same period of advancement. But you will recollect the former case presented us with a bright arterial flush on the face, an active or excited expression of countenance, a very hot skin, and a moderately frequent and full pulse, without any abdominal symptoms. While in the present case you have a darker, more dingy flush, less heat of skin, a quicker but less forcible pulse, and a decidedly dull, indifferent expression, with a constant disposition to drowsiness, and sometimes incoherent talking. You see him falling into a drowsy half-sleeping condition while I am talking by his side. And what does these differences indicate? This is a question of the utmost practical importance. When remarking on the former case, to which I have alluded, it was said that the numerical statistics of M. Louis in regard to the treatment of Pneumonia, were practically not worth a serious examination; simply because they include under the same general head, cases the most diverse in every particular, except the simple existence of pneumonic inflammation. And here you have valid proof of the assertion. Thus in the case of T. Y., we had a strongly marked inflammatory diathesis; or as I should prefer to call it, a high degree of *tonicity* of the solids, with energy of organic action, which required the abstraction of no less than 30 oz of blood, to make a decided impression on the progress of the disease; while here we have altogether the reverse, viz: diminished tonicity with enfeebled organic action, as indicated by the darker hue of the face, the dull and indifferent expression, the early tendency to mental wandering and somnolency, and above all by the frequent and compressible pulse, unaccompanied by any increase in the force of the heart's action. Now, although both patients were attacked with essentially the same disease, and so far as the lapse of time is concerned, they came under our observation at the same period of their progress, yet from the circumstances already detailed, I venture to predict that this patient will not bear the abstraction of the half of 30 oz. of blood without a dangerous degree of prostration.

And the reasons are to be found in the special condition of the solids and fluids of the system, and the grade of activity of the vital properties, at the time of the supervention of disease. You are all aware that exposure to bad air, unwholesome food, and insufficient clothing, as among the poor of cities, in large Hospitals, Jails, Camps, &c., there is induced such a state of the solids and fluids of the system as to strongly favor the occurrence of Typhus Fever, Erysipelas, and indeed, to give whatever disease may occur, a typhoid tendency. So, too, in highly malarious districts, where Intermitting and Remitting fevers prevail during the Summer and Autumn, the solids and fluids undergo a change which modifies, to a greater or less extent, the organic or vital functions. And although this modification is by no means identical with that which constitutes the foundation of true typhus, yet it is evidently accompanied by an impaired tonicity of the solids, and consequently, diminished energy of the functions of *innervation* and *circulation*. And hence any disease, a pneumonic inflammation for instance, occurring in such a system would meet with less of what we may term, vital or organic resistance to its progress; it would pass through its several stages more speedily; the first or congestive stage would be followed by less perfect reaction; the effusion or infiltration in the second stage, would be less plastic and more diffused; and there would be a much earlier failure of innervation, and consequently of capillary circulation. These are the characteristic features of those *Lung Fevers*, *Billious Pneumonias*, *Thphoid Pneumonias*, &c., which are so frequent and fatal in many malarious districts throughout the west, during the winter and spring months. And they are precisely the features which distinguish the case before us from that of T. Y., to whom your attention was directed a few days since. In practice you will find every shade or degree of developement of these features, from the full, healthy degree of tonicity and vital energy exhibited by T. Y., to so great an impairment of these properties, that the first onset of disease meets so little resistance as to scarcely develope reaction in any degree, and the patient is overwhelmed with the most fatal congestion. You, then, who intend to practice medicine, could not be guilty of greater folly than to follow the custom, far too prevalent in the profession, of adopting a special system of treatment of pneumonia or any other acute disease. Such practitioners seem to think it only necessary to determine that pneumonic inflammation exists in any given case, and they bleed, or puke, or purge, according to the system they have adopted, as a matter of course. Hence, while they, during one season, meet with fair success, during another, they loose half their patients before finding out that they are dealing with disease of a different type ; or as I should prefer to term it, with disease occurring in systems with vital properties materially changed.

But you may be ready to ask how the physician is to measure the specific character of the vital properties of any given patient ? I answer by learning to appreciate accurately the true condition of the circulation, both cardiac and capillary, the color and temperature of the skin, and the physiognomy of the patient. Thus an accurate observer would recognize at once, in the dingy color, and dull expression of the face of this patient, both defective innervation and capillary circulation ; which, added to his quick but compressible pulse, his drowsiness, and his lack of acute perception of pain, is abundantly sufficient to mark the specific tendency of the case. I detain you to dwell on these ideas of pathology thus minutely because in the section of country where most of you will practice, it is especially important that you should have the most perfect knowledge of all that class of diseases, of which the case before us serves as a type of one of its most important varieties. And, also, because I wish to destroy that very erroneous idea which considers the whole of *diagnosis* to consist in determining the location, extent, and stage of disease, leaving the determining of its specific character entirely out of view.

*Treatment.* In regard to the treatment of this case, we have the same indications to fulfill or objects to accomplish, as in the same stage of pneumonia occurring under any other circumstances, viz: 1st. To arrest the determination of blood to the diseased part, and thereby prevent the further extension of disease. 2d. To cause the removal of obstruction of the pulmonary tissue now existing in the form of hepatization. 3d. To allay general irritability and sustain the organic actions. But in choosing the means for accomplishing these objects we must have strict reference to that special condition of the system on which we have just been dwelling.

As the patient is young, naturally of a sanguine temperament, the inflammation still extending as indicated by the crepitant râle around its margin, we shall bleed him from the arm notwithstanding the evident impairment of the vital forces. But we shall watch its effects closely, and be ready to sustain the system early and efficiently, if the symptoms which follow require it. Instead of following the bleeding, however, by full nauseating doses of Antimony, or a brisk mercurial cathartic, I shall direct him a powder, composed of Calomel 4 grs. Pulv. Opium 1 gr. Tart. Ant. ¼ gr., to be repeated every three hours through the night, and warm fomentations over the whole abdomen and lower part of the chest. By a very moderate bleeding I shall expect to arrest the determination of blood to the lungs, and procure at least, temporary relief from the oppression of the respiration; while the powders will tend to maintain that relief, by their powerfully diaphoretic and alterative effects, and at the same time soothe and quiet the excessive irritability of the mucous surface of the alimentary canal.

Jan. 11th, 9 o'clock, A. M. Dr. D. remarked that having dwelt so fully on the nature of this case yesterday, he should detain the class only long enough to note carefully the effects of treatment and the progress of the case from day to day, and then occupy their attention with other cases of interest. You perceive, continued the Doctor, scarcely a change in the general symptoms of this patient, except that respiration is less hurried, less painful, and more full. The cough and expectoration is also a little more soft and free. The somnolency, the wandering of mind, the frequency of pulse, and the general expression of the patient, remain about the same as last evening. The skin and tongue, however, are more moist and there have been no evacuations from the bowels. The abstraction of about 12 oz. of blood last evening induced a decided tendency to syncope, with free perspiration and much relief to the breathing, although the pulse neither became more full nor less frequent. The breathing has continued easier through the night, and I am now unable to detect any crepitant râle over any part of the right side ; otherwise the physical signs remain unchanged. I shall direct him a laxative composed of Castor Oil 2 parts, Oil Turpentine 1 part, mixed, ℥ss., and repeat it at the end of four hours if necessary. Discontinue the powders directed yesterday until the bowels have been evacuated, and then renew them at intervals of 4 hours. Also direct immediately a large blister over the infra clavicular region of the right side.

Jan. 12th, 9 o'clock A. M. The first dose of Oil yesterday procured two or three fæcal evacuations of a color and consistence much resembling Tar—the blister has drawn well, and the patient has, since the operation of the oil, taken two of the powders composed of Calomel, Antimony, and Opium. The abdomen is less tympanitic, the breathing still improved, the tongue less red along the edges but still coated and dry on the surface, the expectoration copious but of a redish brown or dirty appearance, urine pretty free but high colored, the skin scarcely above the natural temperature, and the face still somewhat leaden or purplish. The patient has slept much, but sleep disturbed and talkative ; his pulse 105 per minute and easily compressed. Little or no change in the physical signs since yesterday, though the blister renders percussion difficult and unsatisfactory. The whole aspect of the patient shows a moderate degree of improvement since yesterday, but the continued frequency of the pulse, its want of force, the cool state of the skin &c., admonish us to watch closely the vital energies of the patient, and see to it, that in removing the disease we do not allow too great a degree of prostration. The powders must be omitted, and I shall simply direct the following mixture, one teaspoonful every two hours, viz: Compound Honey of Squills, (Hive Syrup,) ℥j, Spts. Nit. Dulc, ℥j, Tinct. Opii et Camph., ℥j., mixed.

Jan. 13th, 9 o'clock A. M. The patient's face is paler, the skin quite cool, the eyes a little more sunken, less drowsy, but still some incoherent talking when left to himself, abdomen not tympanitic; bowels evacuated twice since former visit, and the dischages dark green. The tongue is still dry in the middle, but more moist, and the coat is a little loosened along the margin; the pulse 110, and more feeble than yesterday. The expectoration is copious, less bloody, but streaked with redish purulent matter. There is evidently a less degree of dullness on purcussion over the upper part of the right side, and less bronchophony, but more coarse mucous rhonchus or rattle.

These symptoms present the case in a critical aspect. For while the physical signs, the condition of the abdomen, and the appearance of the tongue, indicate improvement, the continued rapidity and increased feebleness of the pulse, with the appearance of the matter expectorated, show a still further prostration and, threaten the commencement of a diffused suppuration in the pulmonary tissue, which unless promptly arrested, must prove fatal. Hence I shall discontinue the cough mixture directed yesterday, and substitute the following, viz: carbonate of ammonia, 4 grs.; pulverized gum camphor, 1gr.; to be given every four hours; and between each dose 20 drops of the following, viz: tincture of bloodroot, ℥j; tincture opium, ʒj, mixed; and another blister placed on the lower part of the right side immediately.

5 o'clock P. M. Skin warmer, face more flushed, and pulse more forcible. Continue the drops same as before, but direct the powders of camphor and carbonate of ammonia only once in eight hours.

Jan. 14th, 9 o'clock A. M. All the patient's symptoms much improved. The countenance looks brighter; tongue is moist, and the coat becoming detached; expectoration thicker and more yellow; cough less; pulse only ninety-five per minute; and the dullness over the upper part of the right side still less than yesterday. This morning, for the first time, the patient is found lying partly on his left side. Discontinue all medicine except the Tinct. Sanguinaria and Tinct. Opii, mentioned yesterday.

Jan. 15th, 9 o'clock A. M. Patient still doing well. Manifests a little desire for food this morning. Treatment the same.

Jan. 16th, 9 o'clock A. M. Patient steadily improving. Cough and expectoration less, and accompanied with but little sense of soreness in the chest. From this time forward no other treatment was required than the bloodroot mixture three or four times a day, and once a laxative of castor oil. The patient was discharged well on the 22d, twelve days after his admission.

In his concluding remarks on the case, Dr. Davis observed, that every member of the class would now be able to understand what is meant by individual differences of type and tendency in diseases. This patient and T. Y., already several times referred to, were both attacked with plain, unmistakable pneumonic inflammation, but how different the general aspect, progress and treatment of the two; and yet both recovered in nearly the same length of time. They illustrate more forcibly to the mind of the student than a whole volume of written observations, the great supremacy of rational over empirical practice in medicine.

To continue a full report of the Professor's remarks on the following cases would occupy too much space, and hence we shall give only such points as are deemed most useful to the practitioner.

Case 3d. Mr. Jackson, Norwegian, aged twenty years, admitted into the Hospital Jan. 18th, 1851. The patient stated that he had had a paroxysm of intermittent fever every day for several weeks, but during the last four days had had a pretty constant pain in the left hypochondriac region, sometimes very sharp, and always aggravated by coughing or a forced inspiration. His pulse was ninety per minute and moderately full; tongue slightly coated; no appetite; skin dry and warmer than natural; cough frequent, but short and dry. Auscultation and percussion elicited no unnatural sounds, but on extending percussion below the diaphragm over the left hypochondriac region, the whole space occupied by the spleen was found to be very tender, and the organ itself considerably enlarged. The intermittent was thus found, in this case, to be complicated with sub-acute inflammation of the spleen. After pointing out the diagnostic symptoms and signs which distinguish spleenitis from other diseases with which it would be likely to be confounded, it was stated that different views were entertained in regard to the best mode of treating cases of this complicated character.

Some practitioners, fearing the tonic or stimulating qualities of quinine and other anti-periodics, withhold all such remedies until they have subdued the local inflammation, and then they resort to them for the purpose of interrupting the paroxysms of the general disease. Others, viewing the local inflammation simply as a consequence of the repeated congestions induced by the cold stage of the intermittent, tell us to arrest the chills first. In other words, to break up the general disease and the local inflammation will disappear without treatment. Both these views are, to some extent, erroneous; and the practice founded on them will often prove unsuccessful. With the aggravating influence of a protracted chill, recurring every day, or every second day, it is often extremely difficult to subdue a local inflammatory affection; especially in a tissue so distensible and vascular as that of the spleen, and hence a suspension of such chills becomes an essential part of the treatment for removing the disease itself. On the other hand, the influence of the local inflammation on the circulation is such as to favor the recurrence of the chills, and if left unmitigated by direct treatment, will often continue the general disease or convert it into a *remittent* in spite of the most faithful use of anti-periodics. Far the most rational and successful mode is, to direct the treatment both to the general disease and its local complication at the same time. And hence I shall direct, said the Doctor, Sulph. Quinine, 15 grs.; Pillulae Hydrag., 15 grs.; and Pulv. Opii, 1 gr., to be divided into three powders, and one to be taken every four hours, beginning twelve hours before the expected recurrence of the next paroxysm. I shall also cause the affected side to be freely cupped immediately, and the cupping to be followed by warm fomentations.

Jan. 11th. The treatment ordered yesterday has been faithfully carried out. The chill appeared at the usual time to-day, but was much less severe and of shorter duration than usual, and was followed by very little febrile reaction. There is also less tenderness and pain in the region of the spleen. Ordered the same quantity of Sulph. Quinine, Blue Mass and Opium, to be repeated before the time for another chill, and a blister to be applied to the left side.

Jan. 12th. No chill or febrile paroxysm to-day. Blister drawn well, and the pain and tenderness in the side still further diminished. No evacuations from the bowels during the last two days. Ordered Sulph. Magnesia sufficient to move the bowels moderately, and a single dose of Quinine, 5 grs., and Opium, 1 gr., to be given in the morning.

Jan. 13th. The salts yesterday procured two or three free evacuations of a dark green color, and the patient slept well through the night. No return of fever, and at present, only a very slight degree of soreness in the affected side, and much less enlargement of the spleen than at first. From this time forward the patient required no other treatment than a single dose of three grains of quinine every morning, and a continuance of the counter irritation over the region of the spleen. He was discharged well on the eighth day after admission.

Remarks. The propriety of thus directing our treatment to the general and local affections in these cases, at one and the same time, was further illustrated by attention to cases 4th and 5th ; both admitted with intermittent fever of several weeks duration, and one complicated with sub-acute bronchial, and the other hepatic inflammation. Both were promptly cured by the free use of the ordinary anti-periodics, conjointly with local depletion and counter irritation, and the very moderate use of mercurial alteratives. The class were also warned, in connection with these cases, against committing the very common mistake, of supposing an *ague* cured so soon as the paroxysms are suspended ; a mistake which has occasioned many relapses, and consequently done much to destroy the confidence of the community in the efficacy of medicines in the treatment of this disease. It should be remembered that the paroxysms do not constitute the disease, but only the temporary manifestation of the change in the solids and fluids of the system which is induced by the efficient morbific cause ; and which not only precedes the paroxysms, but may also continue for some time after these have ceased. Hence, in all cases, especially of chronic ague, the anti-periodic and tonic remedies should be continued moderately for a considerable time after the chills have ceased to recur, and until a full healthy tone of the system is restored.

Case 6th. Mr. M——, an Irish sailor, aged 40 years, admitted Nov. 29th, 1850. He had been taken several weeks previously with pain, swelling, and lameness of the right hip, accompanied by general febrile symptoms and emaciation. Some ten days since, he presented himself at the College Clinique, where he was examined by Prof. Brainard, and his hip found to be distended with a very large abscess. It was opened by Prof. Brainard just behind the trochanter major, and a large quantity of thick, fetid pus was discharged. He was ordered to use wine and bark internally and keep the affected side at rest. These directions have been followed until he was brought into the Medical Ward of the Hospital to day. In calling the attention of the class to the case, Prof. Davis remarked, that large and unhealthy abscesses originating in the areolar or cellular tissues of the body, had been unusually frequent during the two months which have elapsed since the subsidence of the epidemic cholera in this city. The question of paramount importance in the case before us is, whether the abscess is limited to the soft parts, or has its origin from disease of the hip-joint, on the one side, or the bones of the sacrum on the other. If we seize the foot of the patient and rotate the thigh so as move the head of the femur freely in the socket, or flex the thigh on the pelvis, or bear the head of the bone directly into the bottom of the socket, by firm pressure on the trochanters, no marked pain is produced in the hip-joint; a result hardly compatible with the existence of such a degree of disease of that joint as to give rise to such an abscess. Again, on introducing the probe, it passes upward and backward, deep beneath the glutei muscles, nearly its whole length. But no rough or dead bone can be felt in any direction. Neither is there any special tenderness over any part of the sacrum. These facts, together with the comparatively short time since the affection first manifested itself, lead to the conclusion that the disease is confined to the cellular tissue beneath and between the glutei muscles, and that neither his hip-joint nor the bones of the pelvis are yet involved. But the pale appearance of the tongue and lips, the frequency of the pulse, the thin character of the pus, &c., all indicate a greatly impaired state of the vital forces.

Such being the present condition of the patient, the first and almost the only object of treatment, is to improve the quantity and quality of the blood and the tonicity of the solid tissues. In common parlance, to improve the general health. And how can this be done most effectually? Not by diffusable stimulants, such as wine or brandy and bark; because in this case the properties of *irritability* and nervous susceptibility, are already too active; but by such tonics as improve the strength or tonicity without increasing irritability, and also facilitate the formation of red-corpuscles of the blood. We have here an important practical distinction which is not always duly regarded by the physician. In the true typhus state, the irritability and nervous susceptibility are both diminished, giving an expression of dullness and indifference, not only to the countenance of the patient, but to all his physical or organic actions. In such a state, stimulants are not only well borne, but are often necessary to the maintainance of life. But in the case before us, and in all others like it, we have, it is true, an impoverished condition of the blood; an impaired tonicity, and a too rapid waste of the solids, yet there exists with these, increased irritability and nervous mobility, as indicated by the anxious expression of countenance, the frequency of the pulse, neuralgic pains, and general restlessness. Hence, this patient will be put on the use of the muriated tincture of iron and tincture of conium, equal parts mixed, forty drops three times a day, taken in half a wineglass full of sweetened water. His diet must be simple but nutritious, consisting of plain meat broth, rice, milk and bread. External applications to the affected hip can be of little use at present.

The foregoing treatment was pursued, and the patient began rapidly to improve, and continued to do so for nearly two weeks. The abscess became entirely healed, the swelling had disappeared, and patient improved in flesh and began to walk about the ward with facility. While thus progressing satisfactorily, he began to complain of severe pains, mostly of a lancinating character, in the sacrum, about the tuberosity of the ischium, and along the course of the spermatic cord, extending sometimes to the testicle of that side. The whole surface over these seats of pain became acutely sensitive to the touch, and the evacuations from the bowels greatly aggravated the pain, especially anterior to the crest of the illium and along the course of the spermatic cord. The influence of the passage of fæces along a certain part of the rectum, in aggravating the pain in the cord, was very marked. These symptoms were well calculated to excite, in the minds of some, doubts in regard to the correctness of the former diagnosis; and to cause fears of the formation of another abscess in connection with the bones of the sacrum. But the plainly neuralgic character of the pains—the sensitiveness of the skin over the seats of pain—the little disturbance of the pulse—and, above all, the steady improvement in flesh and strength—led the Doctor to attribute the difficulty to internal hemorrhoïds, complicated with inflammation (or, at least, irritation) of the ganglions of the sacral and lumbar nerves. Hence free cupping over the sacrum and loins, was applied and directed to be repeated daily, for three or four days in succession. This was done, and resulted in entire relief, since which time the patient has remained well, and is doing sailor's duty at this date.

